# αEβ7, α4β7 and α4β1 integrin contributions to T cell distribution in blood, cervix and rectal tissues: Potential implications for HIV transmission

**DOI:** 10.1371/journal.pone.0192482

**Published:** 2018-02-08

**Authors:** Catia T. Perciani, Walter Jaoko, Bashir Farah, Mario A. Ostrowski, Omu Anzala, Kelly S. MacDonald, for the KAVI-ICR Team

**Affiliations:** 1 Department of Immunology, University of Toronto, Toronto, ON, Canada; 2 Kenyan AIDS Vaccine Initiative—Institute of Clinical Research (KAVI-ICR), Nairobi, Kenya; 3 Department of Medical Microbiology, University of Nairobi, Nairobi, Kenya; 4 Keenan Research Centre for Biomedical Science of St. Michael’s Hospital, Toronto, ON, Canada; 5 Section of Infectious Diseases, Department of Internal Medicine, Max Rady College of Medicine, University of Manitoba, Winnipeg, MB, Canada; Emory University School of Medicine, UNITED STATES

## Abstract

Cell surface expression of α4β7, α4β1 and αEβ7 integrins play a key role in T cell distribution. Understanding the contribution of integrins to the density and ratios of CD4^+^: CD4^neg^T cell at the portals of entry for HIV is of fundamental importance for the advance of more effective HIV prevention strategies. We therefore set out to characterize and compare the expression of α4β7, α4β1 and αEβ7 integrins on systemic, cervical and rectal CD4^+^ and CD4^neg^T cells isolated from a cohort of healthy Kenyan women at low risk for sexually transmitted infections (STI) (n = 45). Here we show that blood and cervix were enriched in α4^+^β1^+^CD4^+^T cells and α4^+^β7^hi^CD4^+^T cells, whereas the rectum had an equal frequency of α4^+^β7^hi^CD4^+^T cells and αE^+^β7^hi^CD4^+^T cells. Most cervical and rectal αE^+^β7^hi^CD4^+^T cells expressed CCR5 as well as CD69. Interestingly, αEβ7 was the predominant integrin expressed by CD4^neg^T cells in both mucosal sites, outnumbering αE^+^β7^hi^CD4^+^T cells approximately 2-fold in the cervix and 7-fold in the rectum. The majority of αE^+^β7^hi^CD4^neg^T cells expressed CD69 at the mucosa. Taken together, our results show unique tissue-specific patterns of integrin expression. These results can help in guiding vaccine design and also the use of therapeutically targeting integrin adhesion as a means to preventing HIV.

## Introduction

Most HIV transmission globally occurs through sexual intercourse. Scrutinizing the events associated with the influx of activated CCR5^+^CD4^+^T cells into the genital and gut mucosa and the maintenance of a pool of HIV-specific effector memory CD8^+^T cells at the portal of entry to HIV can inform HIV vaccine and therapy design. Integrins are αβ heterodimeric, transmembrane proteins that among other functions, direct cell trafficking and retention at various anatomical sites [1]. Among the 24 αβ integrin pairs identified to date, three of them are especially important for T cell localization: α4β7, αEβ7 and α4β1. α4β7 integrin binds predominantly to MAdCAM-1 (mucosal addressin cell adhesion molecule-1), a molecule expressed on endothelial cells of the gastrointestinal and genital tract, and it is well known as a gut-homing marker [2]. αEβ7 binds to E-cadherin and plays a role on T cell retention in epithelial tissues such as skin and gut [3, 4]. α4β1 integrin, also named VLA-4 (very late antigen-4), is expressed on monocytes and lymphocytes, but in contrast to the first two integrins is also expressed on many other cell types. α4β1 binds to VCAM-1 (vascular cell adhesion protein-1) and can direct cell migration to a diverse set of sites, including the genital tract, gut, lungs and brain.

Studies have demonstrated that CD4^+^T cells expressing α4β7 and α4β1 are more susceptible to HIV infection. CD4^+^T cells harboring α4β7 were preferentially targeted during HIV/SIV infection [5, 6]. High expression of α4β7 in memory CD4^+^T cells has been shown to correlate with increased susceptibility to rectal SIV infection and are associated with higher viral loads in macaques [7, 8]. Increased availability of α4β7^+^CD4^+^T cells in the vaginal tissue has been associated with an increased risk of SHIV acquisition [9]. In humans, the frequency of α4β7^+^CD4^+^T cells in peripheral blood has been shown to be associated with increased rates of HIV infection and HIV clinical outcomes [10]. Additionally, α4β1-expressing CD4^+^T cells isolated from cervix were shown to be preferentially infected with HIV R5-pseudovirus in an *in vitro* assay [11].

The association of enhanced HIV susceptibility with α4β7^+^CD4^+^T cells availability encouraged the investigation of targeting α4β7 with humanized anti-α4β7 monoclonal antibodies (mAbs) on SIV/HIV infection. Anti-α4β7 mAbs have been used in humans to treat ulcerative colitis and Crohn’s disease [12, 13]. Administration of anti-α4β7 mAb in a non-human primate (NHP) model challenged with SIV_mac251_ intravaginally had a significant impact on decreasing SIV acquisition and delaying disease progression [14]. More recently Byrareddy et al (2016) showed that a regimen of anti-retroviral therapy (ART) combined with anti-α4β7 mAb was able to suppress viral load in rhesus macaques infected with SIV_mac239_ with no viral rebound observed even after both therapies were stopped [15]. The mechanisms by which anti-α4β7 mAb have conferred protection remains elusive.

Conversely, there is growing evidence that the formation and maintenance of a pool of tissue resident memory T (T_RM_) cells can play a pivotal role in mounting rapid recall responses [16, 17] and generation of an antiviral state [18, 19]. Despite the absence of definitive markers of T_RM_ cells, there is an agreement about the importance of CD103 (αE) expression in this population. Although most of the studies discuss T_RM_ as CD8^+^T cells, CD4^+^T cells also persist at the tissue as T_RM_ cells [20, 21]. The role of αEβ7 as an adhesion molecule in this context has been under-explored and invites further investigation especially in humans.

In this study, we characterized the frequency of CD4^+^ and CD4^neg^T cells expressing αE^+^β7^hi^, α4^+^β7^hi,^ α4^int^β7^int^ and α4^+^β1^+^ in blood, cervix and rectum of healthy Kenyan women and also their co-expression with the early activation marker CD69. The frequency of integrin expressing-CD4^+^T cells co-expressing CCR5 in these sites were also a focus of analysis.

Our work reveals that cervical and rectal αE^+^β7^hi^CD4^+^T cells displayed the highest expression of CCR5 and CD69 when compared to CD4^+^T cells expressing the other integrins or compared to α4^-^β7^-^CD4^+^T cells. Analysis of integrin expressions on CD4^neg^T cells revealed that αEβ7 is particularly important for the distribution of this cell type in mucosal sites potentially serving as a key integrin that determines and maintains a protective frontline pool of cells at the site of infection. We have defined and detailed the compartmentalization of integrin expression on T cell subsets in directly relevant human tissue i.e. the sites of HIV entry and believe this work will facilitate the development of optimized vaccines and therapeutics that take into account the diversities of mucosal tissues involved in HIV susceptibility and protection against infection.

## Material and methods

### Clinical specimens

Baseline samples collected from women enrolled at KAVI-VZV-001 trial (n = 45) were included in this study. The participants enrolled at the study aged 26 (21.5–30.5) years (median, IQR), were seronegative for HIV-1 and HIV-2 and determined non-pregnant. Written informed consent was obtained for all subjects participating in the trial. This study was approved by KNH/UON ERC (Reference Number KNH-ERC/A/352), University of Toronto REB (Protocol Number 31043) and by Kenyan Pharmacy and Poisons Board (Reference Number PPB/ECCT/15/01/02/2015). This study was conducted and the data generated recorded and reported in accordance with the ICH Guidelines for Good Clinical Practice, regulatory requirements and the Declaration of Helsinki.

### Isolation of Peripheral Blood Mononuclear Cells (PBMCs), cervical cells, and rectal cells

BD Vacultainer® sodium heparin tubes, cytobrushes (Digene®, Qiagen), and Sarrat disposable forceps (STE1500, Stericom) were used for the collection of blood, cervical cells and rectal cells respectively as previously described [22]. PBMCs were isolated by gradient using Histopaque^®^- 1077 Hybri-Max (Sigma-Aldrich). Cervical cells were mechanically isolated from the cytobrushes and rectal cells were isolated from 9 punch-biopsies using 2 cycles of digestion with collagenase type II (Sigma) under agitation at 37°C. All samples were analyzed fresh.

### Multicolor flow cytometric analysis

PBMCs, cervical cells and rectal cells were stained with pre-determined concentrations of antibodies directed against CD3 (clone SK7) (eBioscience), CD4 (clone SK3) (BD Horizon), CCR5 (clone 2D7) (BD Horizon), CD69 (clone FN50) (BD Pharmingen), CD49d (clone 9F10) (eBioscience), and β7 (clone FIB504) (BD Pharmingen). Dead cells were marked using LIVE/DEAD Far Red Cell Stain Kit (Invitrogen). Some samples were also stained with antibodies against CD103 (clone Ber-ACT8) (BioLegend). An LSRII flow cytometer driven by the DiVa software package (BD Biosciences) was used to acquire the samples. Analysis was performed on FlowJo v10.1 software (FlowJo, LLC, USA).

### Statistical analysis

Spearman’s correlation (r_s_) and Friedman test followed by Wilcoxon Signed Rank test were used to compare the variables.

P-values were adjusted for multiple-comparisons using a step-down procedure. Statistical analysis were performed using IBM^®^SPSS^®^Statistics. Graphs were generated using Prism 6 (GraphPad, USA) software. A P-value of <0.05 was considered to be statistically significant, throughout the manuscript *P < 0.05 **P < 0.01 ***P < 0.001 ****P < 0.0001 and relevant statistical tests are specified in each figure legend.

## Results

### Anti-α4 and anti-β7 co-staining allows for the identification of αEβ7 integrin populations within T cells

**Fig 1:**
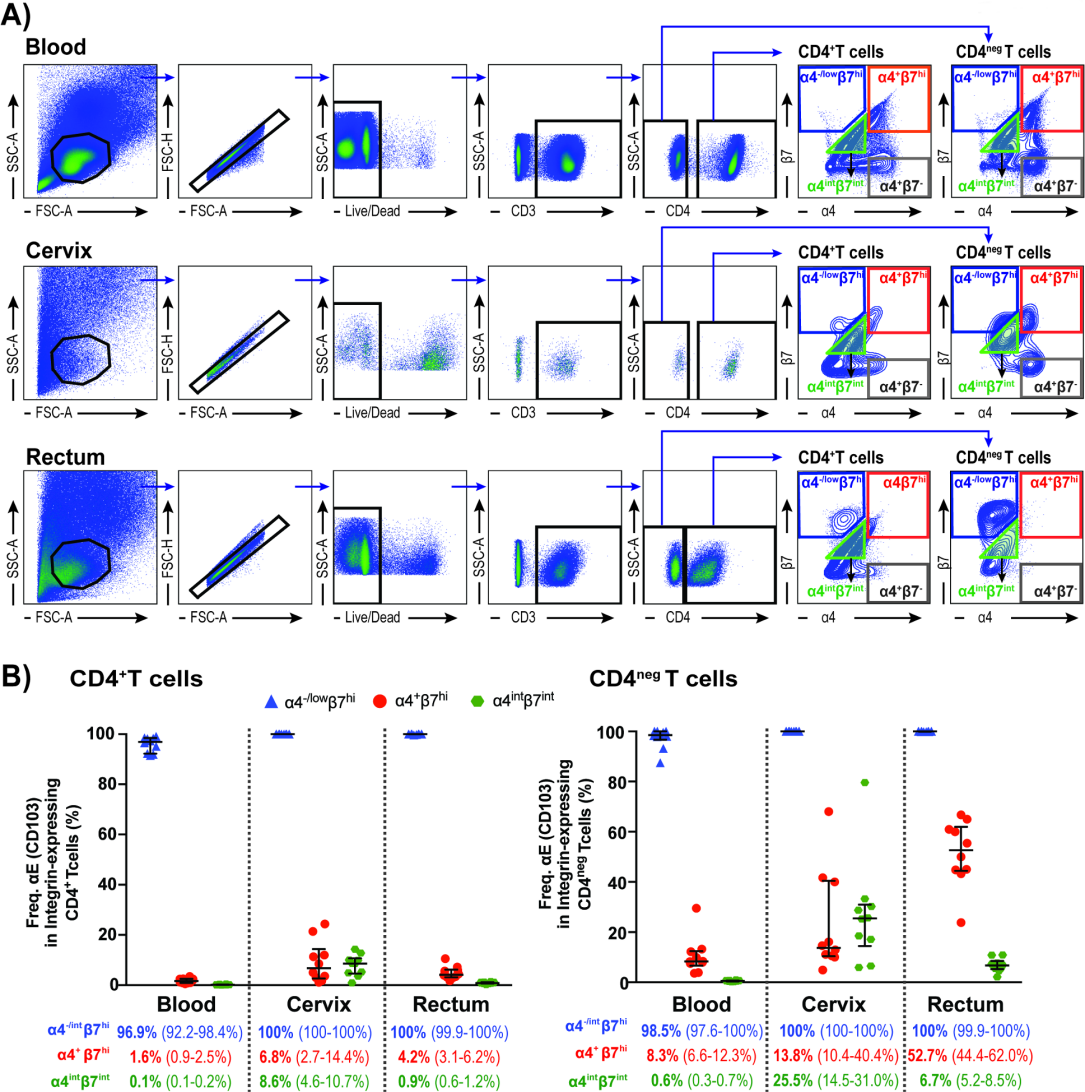
Anti-α4 and anti- β7 co-staining as means to the identification of αE^+^β7^hi^ T cell population. (A) Representative flow cytometry plots for the identification of α4^-/neg^β7^hi^, α4^+^β7^hi^, α4^int^β7^int^ and α4^+^β7^-^T cell populations in blood, cervix and rectum; (B) Frequency of α4^-/low^β7^hi^, α4^int^β7^int^ and α4^+^β7^hi^ on CD4^+^ and CD4^neg^T cells expressing αE. Data from 10 female subjects presented as median and interquartile range (IQR).

Peripheral blood, cervical cytobrushes and rectal biopsies were collected from healthy Kenyan women enrolled in the KAVI-VZV 001 study (ClinicalTrials.gov Identifier: NCT02514018)[22]. CD4^+^T cells and CD4^neg^T cells isolated from these tissues were analyzed for their expression of α4 and β7 integrins. Our gating strategy for this analysis is shown in Fig 1A. Mucosal T cells, especially rectal T cells, showed two distinct β7^hi^ populations based on their level of α4 expression (Fig 1A). As the β7 chain can pair with α4 or αE, we tested these two distinct β7^hi^ populations as well as the α4^int^β7^int^ and α4^+^β7^-^ populations for the expression of αE (CD103) (S1 Fig). Blood, cervical and rectal samples isolated from ten volunteers were stained with anti-αE (anti-CD103) antibody. We observed that both α4^-/low^β7^hi^CD4^+^T cells and α4^-/low^β7^hi^CD4^neg^T cells were positive for αE in the three tissues analyzed (Fig 1C). We also observed that a subset of α4^+^β7^hi^T cells and of α4^int^β7^int^T cells co-expressed αE (Fig 1C) in agreement with previous reports [23, 24]. We also analyzed the expression of αE in the population expressing intermediate levels of α4 and β7 by dividing it into two subsets, I and II (Panel B in S1 Fig). Subset I, comprising the cells expressing higher levels of β7, exhibited increased expression of αE compared to subset II (Panel C in S1 Fig). We also observed a strong positive correlation between the mean fluorescence intensity (MFI), a measure of integrin density/cell, for αE and β7 on CD4^+^T cells in blood (r_s_ = 0.74), cervix (r_s_ = 0.79) and rectum (r_s_ = 0.84), as well as on CD4^neg^T cells in blood (r_s_ = 0.83) and rectum (r_s_ = 0.96) (S2 Fig).

As it has been shown that α4^+^β7^-^CD4^+^T cells express β1 [11], by staining cells with antibodies against α4 and β7 we could confidently identify three important integrins involved in migration and retention of T cell populations: α4β7, αEβ7 and α4β1.

β7^hi^ has previously been used for the identification of α4β7^+^ in blood CD4+T cells [25] and has been shown to be a predictor of HIV outcome in humans [10]. Using our gating strategy for identification, we next determined the density of β7 in the three integrin-expressing T cells that carried β7 in its heterodimeric forms: αE^+^β7^hi^, α4^+^β7^hi^, and α4^int^β7^int^. We observed that the density of β7 was higher in α4^+^β7^hi^CD4^+^T cells isolated from blood and cervix and in αE^+^β7^hi^CD4^+^T cells isolated from rectum than in the other integrin-expressing CD4^+^T cells (S3 Fig). With the exception of blood, in which α4^+^β7^hi^ and αE^+^β7^hi^ showed equivalent β7 densities, a similar profile was observed in CD4^neg^T cells (S3 Fig). As shown in S3 Fig, α4^int^β7^int^ population exhibited lower α4 and αE densities when compared to α4^+^β7^hi^ and αE^+^β7^hi^, respectively (S3 Fig). The densities of β7, α4, and αE were also analyzed on α4 and/or αE expressing T cells (S4 Fig). We observed that α4^+^αE^+^ T cells showed higher β7 density when compared to cells carrying only α4 or αE (S4 Fig). In blood, T cells co-expressing α4 and αE were shown to have a higher density of α4 when compared to cells carrying only one of the integrins (S4 Fig). The same trend was observed in blood for the density of αE (S4 Fig). In contrast, mucosal T cells co-expressing α4 and αE showed lower α4 MFI then α4^+^αE^-^ T cells. Interestingly, αE MFI in α4^+^αE^+^ T cells were equivalent to α4^-^αE^+^ T cells in both cervix and blood and was significantly higher in rectal α4^+^αE^+^ T cells (S4 Fig).

### The highest levels of CCR5 and CD69 expressions are observed in the αE^+^β7^hi^CD4^+^T cells at the mucosa

**Fig 2:**
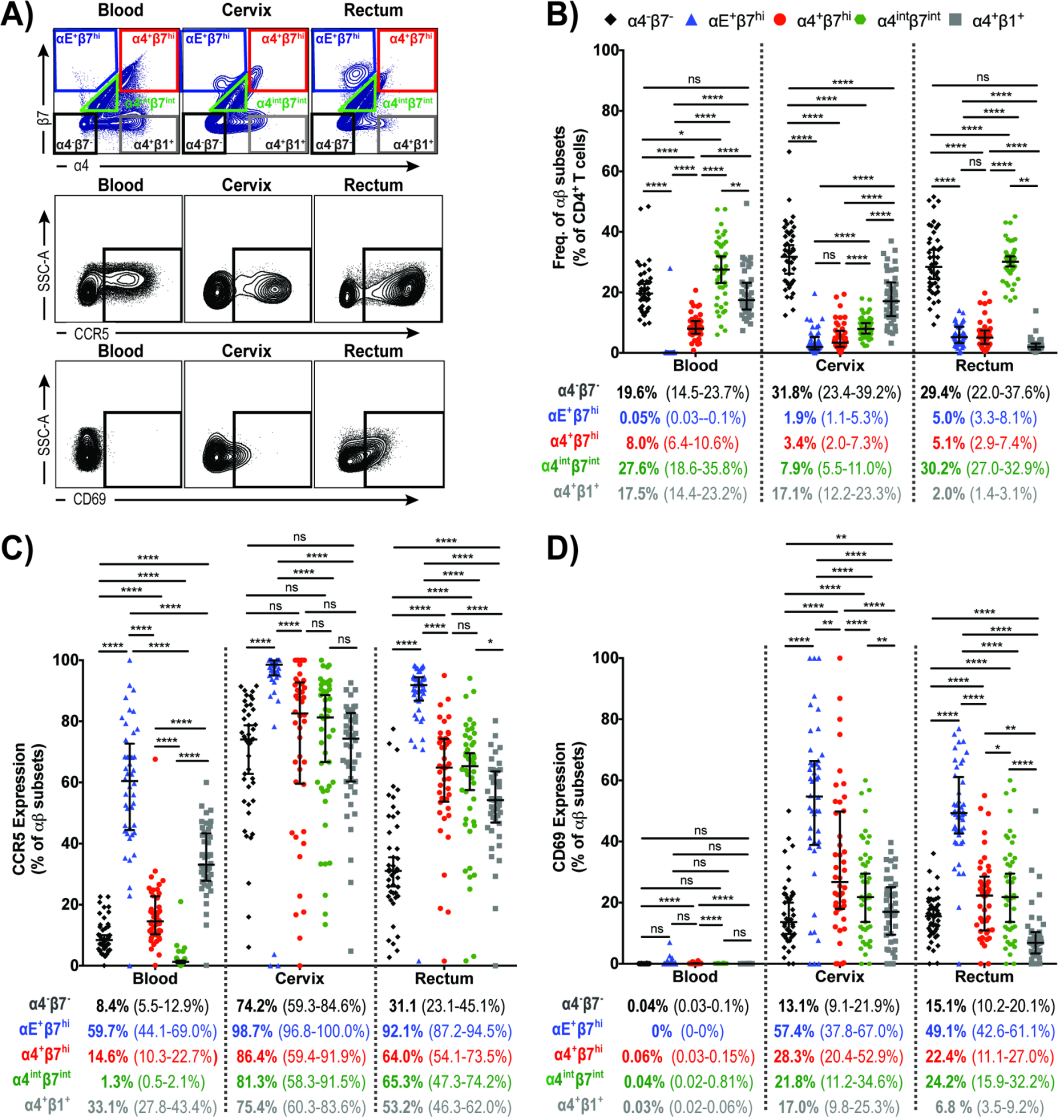
Integrin-expressing CD4^+^T cells isolated from blood, cervix and rectum and their co-expression with CCR5 and CD69. (A) Representative flow cytometry plots for the identification of α4^-^β7^-^, αE^+^β7^hi^, α4^+^β7^hi^, α4^int^β7^int^ α4^+^β1^+^, CCR5^+^ and CD69^+^ on CD4^+^T cell populations in blood, cervix and rectum; (B) α4^-^β7^-^CD4^+^T cells (black), αE^+^β7^hi^ (blue), α4^+^β7^hi^ (red), α4^int^β7^int^ (green) and α4^+^β1^+^ (gray) expression on CD4^+^T cells isolated from blood, cervix and rectum. (C) Frequency of CCR5-expressing cells on α4^-^β7^-^CD4^+^T cells (black), αE^+^β7^hi^CD4^+^T cells (blue), α4^+^β7^hi^CD4^+^T cells (red), α4^int^β7^int^ CD4^+^T cells (green) and α4^+^β1^hi^CD4^+^T cells (gray). (D) Frequency of CD69-expressing cells on α4^-^β7^-^CD4^+^T cells (black), αE^+^β7^hi^CD4^+^T cells (blue), α4^+^β7^hi^CD4^+^T cells (red), α4^int^β7^int^ CD4^+^T cells (green) and α4^+^β1^+^CD4^+^T cells (gray). Data from 45 female subjects presented as median (IQR). *P < 0.05 **P < 0.01 ***P < 0.001 ****P < 0.0001, as calculated by Friedman Test, followed by Wilcoxon signed rank-test, and adjusted for multiple comparisons using step-down procedure.

Representative flow cytometry plots for the identification of αβ subsets within CD4^+^T cells populations as well as CCR5^+^ and CD69^+^CD4^+^ T cell populations in blood, cervix and rectum are shown in Fig 2A. We observed that systemic and cervical CD4^+^T cells predominantly expressed α4^+^β1^+^ (17.5% and 17.1% of total CD4^+^T cells, respectively) followed by α4^+^β7^hi^ (8.0% in blood and 3.4% in cervix) (Fig 2B). As expected, CD4^+^T cells expressing αE^+^β7^hi^ were very rare (0.05%) in blood. Interestingly, we observed that αE^+^β7^hi^CD4^+^T cells in cervix were present at comparable level to the α4^+^β7^hi^CD4^+^T cell population. Similarly, in the rectum, the frequency of α4^+^β7^hi^CD4^+^T cells and αE^+^β7^hi^CD4^+^T cells were equivalent (approximately 5%), although in this tissue α4^+^β1^+^CD4^+^T cells were shown to be significantly less frequent (Fig 2B). Approximately 30% of blood and rectal CD4^+^T cells displayed intermediate expression of α4 and β7 (α4^int^β7^int^) (Fig 2B), a population that in these tissues were shown to minimally co-express αE (< 1%). The frequency of α4^int^β7^int^CD4^+^T cell population in cervix was 7.9%, from which approximately 10% are expected to co-express αE (Figs 1B and 2B). It is worth noting that approximately one-quarter of the CD4^+^Tcells in blood, cervix and rectum were negative for these three integrins (Fig 2B).

We next sought to compare the frequency of integrin-expressing CD4^+^T cells harboring the HIV co-receptor CCR5. We observed that overall αE^+^β7^hi^, α4^+^β7^hi^, α4^int^β7^int^ and α4^+^β1^+^CD4^+^T cells at the mucosa often co-expressed CCR5, with strikingly more than 90% of αE^+^β7^hi^CD4^+^T cells in both cervix and rectum expressing CCR5 (Fig 2C). The frequency of α4^+^β7^hi^CD4^+^T cells and α4^+^β1^+^CD4^+^T cells expressing CCR5 at the rectal tissue was 64% and 53%, respectively (Fig 2C). At the mucosal sites, the frequency of CCR5-expressing α4^int^β7^int^ CD4^+^T cells was similar to the ones observed for α4^+^β7^hi^CD4^+^T cells (Fig 2C). With exception of blood α4^int^β7^int^ CD4^+^T cells, integrin-expressing CD4^+^T cells showed higher CCR5 co-expression than α4^-^β1^-^CD4^+^T cells in the three tissues studied, yet the CCR5 expression on α4^-^β1^-^CD4^+^T cells was surprisingly high in cervix when compared to the other tissues (Fig 2C). Next, we analyzed the expression of CD69 on integrin-expressing CD4^+^T cells isolated from blood, cervix and rectum. Although CD69 expression has been used as a marker of early cell activation, more recently it has been shown to exercise functions related to tissue residence even in the absence of cell activation [26]. CD69 can physically interact to the Sphingosine-1-Phosphate Receptor-1 (S1P_1_) leading to its degradation, and prolonging T cell retention in the tissue, potentially assisting in T_RM_ formation [27, 28]. Mucosal αE^+^β7^hi^CD4^+^T cells presented the highest levels of CD69 expression when compared to the other integrin subtypes and to the α4^-^β7^-^CD4^+^T cell population (Fig 2D).

### CD4^neg^T cells in both cervix and rectum abundantly express αEβ7

**Fig 3:**
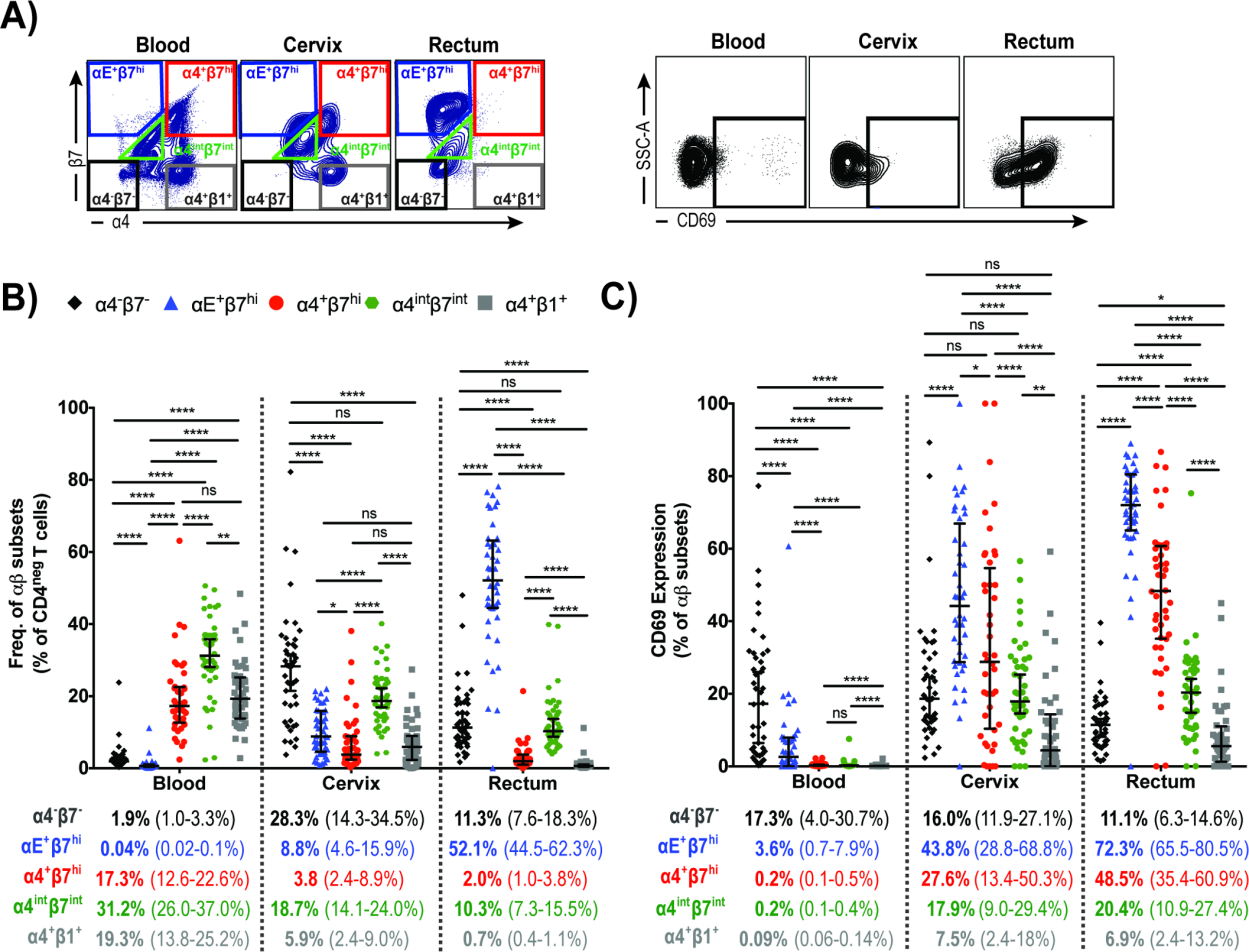
Integrin-expressing CD4^neg^T cells isolated from blood, cervix and rectum and their co-expression with CD69. (A) Representative flow cytometry plots for the identification of α4^-^β7^-^, αE^+^β7^hi^, α4^+^β7^hi^, α4^int^β7^int^, α4^+^β1^+^, and CD69^+^ on CD4^neg^T cell populations in blood, cervix and rectum; (B) αE^+^β7^hi^ (blue), α4^+^β7^hi^ (red), α4^int^β7^int^ (green) and α4^+^β1^+^ (gray) expression on CD4^neg^T cells isolated from blood, cervix and rectum. (C) Frequency of CD69-expressing cells on α4^-^β7^-^CD4^neg^T cells (black), αE^+^β7^hi^CD4^neg^T cells (blue), α4^+^β7^hi^CD4^neg^T cells (red), α4^int^β7^int^CD4^neg^T cells (green) and α4^+^β1^+^CD4^neg^T cells (gray). Data from 45 female subjects presented as median (IQR). *P < 0.05 **P < 0.01 ***P < 0.001 ****P < 0.0001, as calculated by Friedman Test, followed by Wilcoxon signed rank-test, and adjusted for multiple comparisons using step-down procedure.

Here we examined the expression of αEβ7, α4β7 and α4β1 integrins on CD4^neg^T cells from blood, cervix and rectal tissue as well as their co-expression with CD69 (representative flow cytometry plots for their identification are shown in Fig 3A). As observed in Fig 3B, αE^+^β7^hi^ was predominantly expressed on both cervical and rectal CD4^neg^T cells (8.8% and 52.1%, respectively) and practically absent in blood CD4^neg^T cells (Fig 3B). The frequency of CD4^neg^T cells expressing α4^+^β7^hi^ or α4^+^β1^+^ was comparable in blood (17.3% and 19.3%, respectively). In cervix the frequency of α4^+^β7^hi^CD4^neg^T cells, α4^+^β1^+^CD4^neg^T cells and αE^+^β7^hi^CD4^neg^T cells were all very similar (Fig 3B), with predominance of α4^int^β7^int^ in this tissue. Rectal CD4^neg^T cells exhibited a unique integrin expression profile, with approximately 50% of the cells harboring αE^+^β7^hi^. Although in a significantly lower level than αE^+^β7^hi^, CD4^neg^T cells expressing α4^int^β7^int^, α4^+^β7^hi^ and α4^+^β1^+^ were also detected in rectum (10.3%, 2.0% and 0.7%, respectively) (Fig 3B).

When we analyzed the level of CD69 expression on αE^+^β7^hi^CD4^neg^T cells, we observed that 43.8% of cervical αE^+^β7^hi^CD4^neg^T cells and 76% of rectal αE^+^β7^hi^CD4^neg^T cells expressed CD69, indicating that most of these cells have a low migratory capability (Fig 3C) potentially been identified as T_RM_ cells. CD69 expression was also high in α4^+^β7^hi^CD4^neg^T cells isolated from both cervix and rectum (27.6% and 48.5% respectively), indicating reduced circulatory potential (Fig 3C).

### The frequency of CD4^+^T cells expressing α4^+^β7^hi^ correlated across blood, cervix and rectum

**Fig 4:**
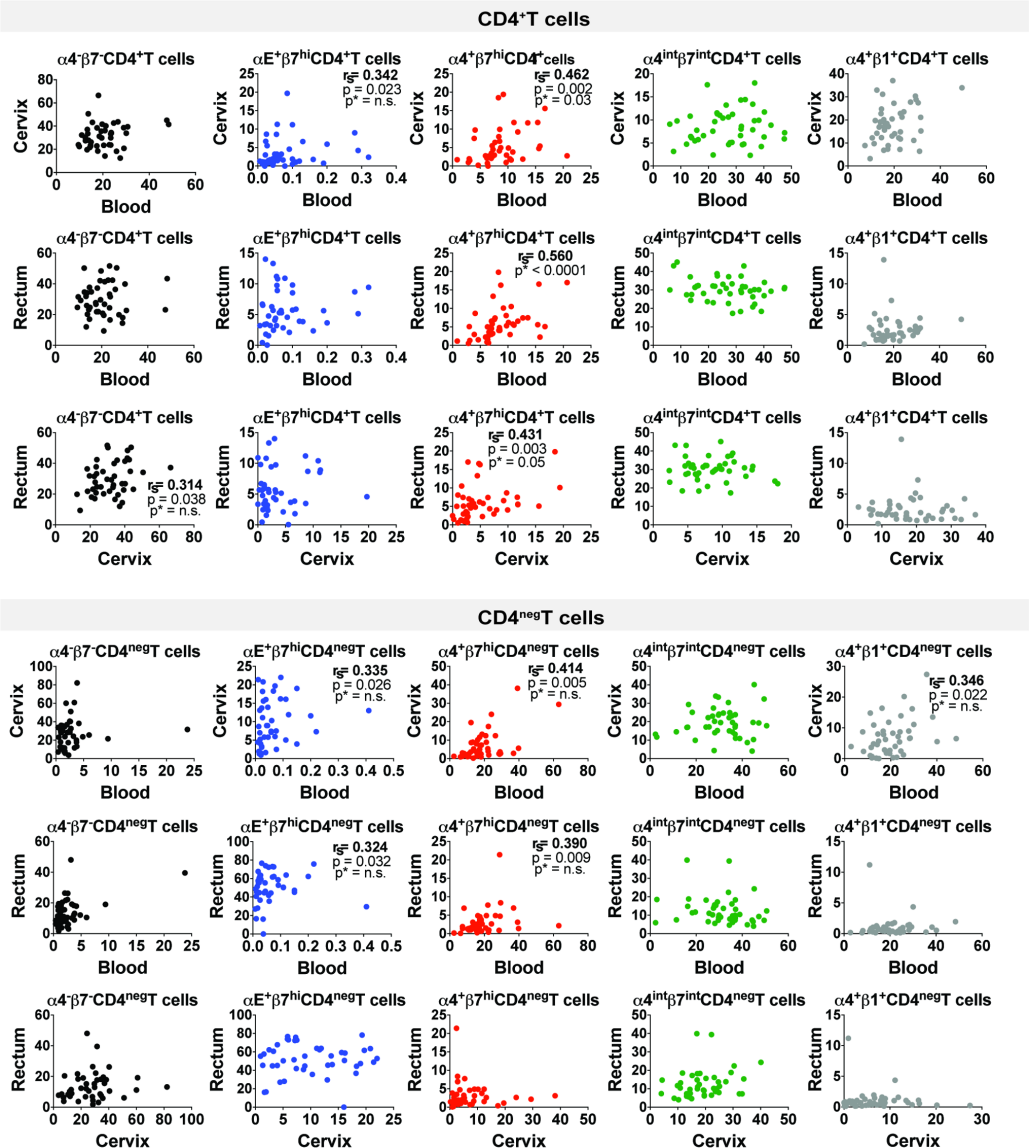
Correlations of integrin-expressing cells between tissues. Graphs display Spearman’s correlation (r_s_), and both unadjusted and adjusted p values (n = 10). P values adjusted for multiple comparisons are marked with asterisks (p*).

We further evaluated whether the frequency of integrin-expressing T cells in one tissue could be used as predictor of their expression in another tissue. Among all the integrin subsets studied here (α4^-^β7^-^, αE^+^β7^hi^, α4^+^β7^hi^, α4^int^β7^int^, and α4^+^β1^+^) in CD4^+^ and in CD4^neg^T cells, only α4^+^β7^hi^ CD4^+^T cells correlated across the three tissues after adjusting for multiple comparisons (Fig 4). There was also a positive correlation between the frequencies of α4^+^β7^hi^ CD4^neg^T cells in blood and cervix (r_s_ = 0.41) and in rectum and blood (r_s_ = 0.39); as well as between the frequencies of αE^+^β7^hi^ T cells in blood and cervix (r_s_ = 0.34), αE^+^β7^hi^ CD4^neg^T cells in blood and rectum (r_s_ = 0.32), and α4^+^β1^+^ CD4^neg^T cells in blood and cervix (r_s_ = 0.35), however these correlations showed not to be significant after adjusting for multiple comparisons.

### Integrin contributions for the CD4^neg^:CD4^+^ T cell ratio in blood, cervix and rectum

**Fig 5:**
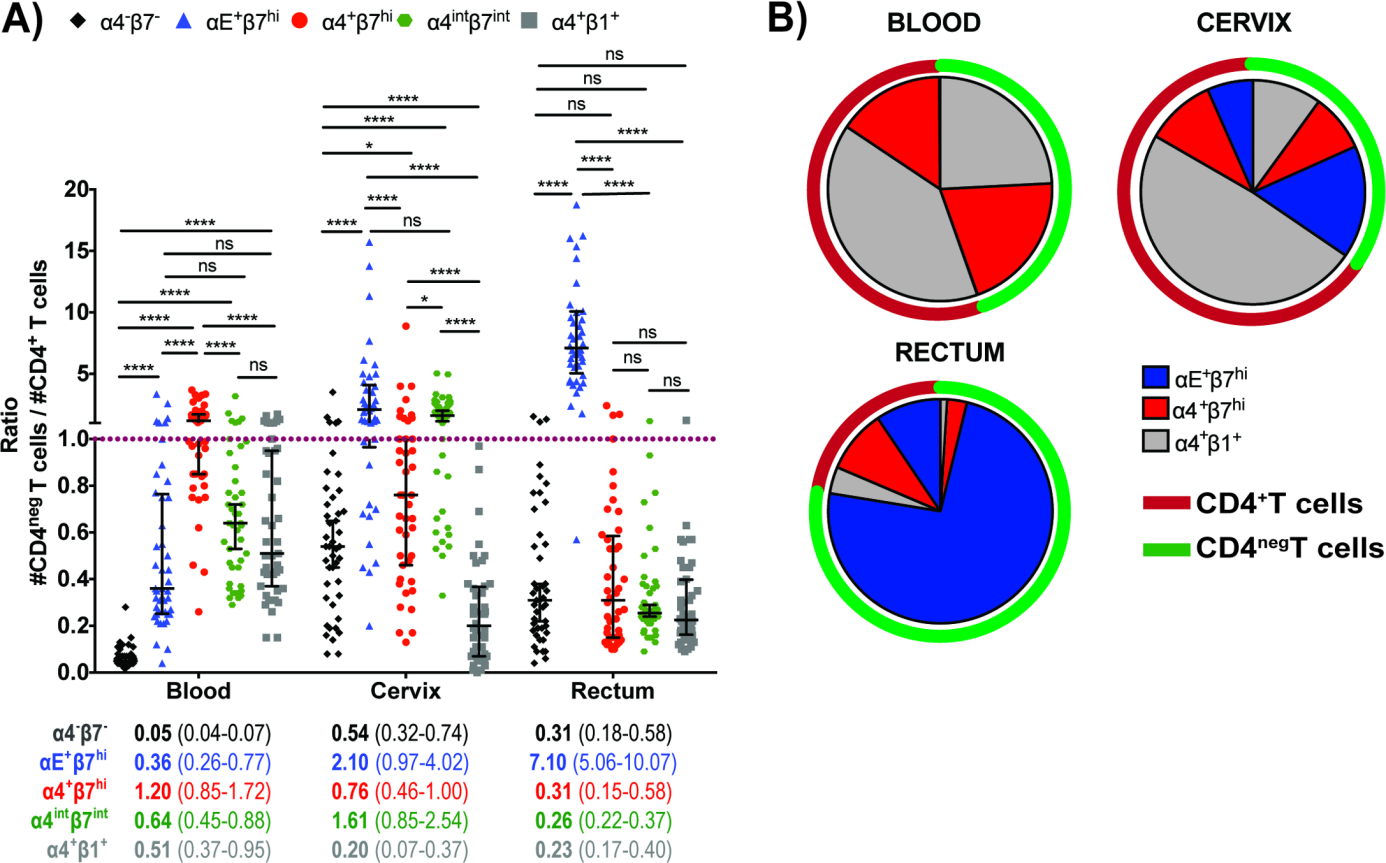
Integrin-expressing CD4^+^ and CD4^neg^T cell densities in the blood, cervix and rectum. (A) CD4^neg^:CD4^+^ ratio of all CD3^+^cells expressing α4^-^β7^-^, αE^+^β7^hi^, α4^+^β7^hi^, α4^int^β7^int^ or α4^+^β1^+^ in blood, cervix and rectum. (B) The densities of αE^+^β7^hi^, α4^+^β7^hi^ and α4^+^β1^+^T cells in this pie charts were drawn based on integrin-expressing T cells in each tissue and does not account for the total density of T cells in each site. Data from 45 female subjects presented as median (IQR). *P < 0.05 **P < 0.01 ***P < 0.001 ****P < 0.0001, as calculated by Friedman Test, followed by Wilcoxon signed rank-test, and adjusted for multiple comparisons using step-down procedure.

The presence of large numbers of effector CD8^+^T cells in proximity to target cells could potentially prevent or control infection locally. Unfortunately there is still a lack of information about the importance of integrins for both CD4^+^ and CD8^+^T cells positioned at the mucosa, especially at the human genital mucosa. We therefore set out to explore the CD4^neg^:CD4^+^ ratio of all CD3^+^cells expressing each of the three integrins studied here. Unlike α4^-^β7^-^, α4^+^β1^+^, and α4^+^β7^hi^, αE^+^β7^hi^ was predominantly expressed on mucosal CD4^neg^T cells, with a CD4^neg^:CD4^+^ ratio of 2.1 in cervix and 7.1 in the rectum (Fig 5A) (p<0.0001). At the cervix, α4^int^β7^int^ was also shown to be expressed more in CD4^neg^T cells, with a CD4^neg^:CD4^+^ ratio of 1.6 (Fig 5A). The contribution of α4^+^β1^+^, α4^+^β7^hi^, and αE^+^β7^hi^ to integrin-expressing T cell densities in blood, cervix, and rectum are shown in Fig 5B. Circulating CD4^+^T cells expressed predominantly α4^+^β1^+^ integrin while circulating CD4^neg^T cells equally expressed α4^+^β7^hi^ or α4^+^β1^+^. The integrin expression on both CD4^+^ and CD4^neg^T cells in the cervix was more heterogeneous than in the other tissues analyzed, with a predominance of α4^+^β1^+^CD4^+^T cells and αE^+^β7^hi^CD4^neg^T cells in this tissue. Conversely, the rectal tissue showed an unparalleled T cell composition based on the integrin expression. CD4^+^T cells expressing high levels of αE^+^β7^hi^ or α4^+^β7^hi^ were equally frequent in the rectum, while, CD4^neg^T cells in rectum frequently expressed αE^+^β7^hi^, with a minimal presence of α4^+^β7^hi^ or α4^+^β1^+^CD4^neg^T cells (Fig 5B).

## Discussion

The distribution of T cells in mucosal tissues directly impacts on protection against pathogens as well as disease outcome. The availability of activated CCR5^+^CD4^+^ T cells is known to increase susceptibility to HIV infection [29, 30]. In addition, CD4^+^T cell depletion in HIV infected individuals leads to a pronounced impairment of their gut-associated lymphoid tissue (GALT) function which is not reversed even after viral suppression with ART [31–34]. Therefore, understanding the distinct integrin expression that mediated cell migration and retention may assist in targeting HIV at the mucosa and restore gut immunity.

In this study we comprehensively assessed the frequency of CD4^+^ and CD4^neg^T cells expressing the integrins αEβ7, α4β7 and α4β1 in blood and in two major sites for HIV infection, cervix and rectum. Importantly, all of these findings are based on the *ex vivo* determinations of these integrins on HIV susceptible CD4^+^T cells and other T cell populations at the sites of HIV entry, significantly enhancing the importance of our findings to establish new strategies aimed at the treatment and prevention of HIV infection.

Here we demonstrated that it is correct to identify the α4^-/low^β7^hi^ population as αE^+^β7^hi^, encouraging its analysis even when the number of markers that can be included in the flow cytometric panels and the costs of reagents may pose a challenge. Additionally, this observation proves that it is incorrect to identify all β7^hi^ populations as α4^+^β7^hi^ for cells isolated from mucosal tissues.

Admittedly the complexities, cost and potential invasiveness associated with mucosal sampling and analyses have confined most HIV clinical trials to base their conclusions on parameters from peripheral blood. However, as we have shown, blood phenotypic analysis is not always reflective of cell phenotypes at the critically relevant mucosal sites of initial viral challenge, infection and multiplication. Here, we demonstrated that CD4^+^ and CD4^neg^T cells differentially exhibit various integrin-populations depending on their location. It has been shown that the frequency of α4^+^β7^hi^ in blood positively correlated with the frequency of these cells in cervix [10]. Here we also observed a positive correlation between α4^+^β7^hi^ on CD4^+^T cells between these tissues and were able to demonstrate that the frequency of this population also correlated between blood and rectum, and between cervix and rectum (Fig 4). For all the other αβ subsets studied here, no significant correlation was observed across the three tissues after adjusting for multiple comparisons.

It has previously been shown that in black men who have sex with men (MSM), a population with higher HIV incidence rates, exhibited significantly higher density of the β7 integrin on blood CD4^+^T cells than MSM of other race/ethnicities [35]. In a recent study, Sivro et al (2018) demonstrated that in blood the frequency of β7^hi^ CD4^+^T cells prior to infection, but not β7^int^ or β7^neg^, was correlated with set point viral load post-infection [10]. In our study we showed that the density of β7 was higher in α4^+^β7^hi^CD4^+^T cells isolated from blood and cervix and in αE^+^β7^hi^CD4^+^T cells isolated from rectum (S3 Fig). Additionally, a subset of T cells double positive for α4 and αE also displayed higher β7 density when compared to cells carrying only α4 or αE (S4 Fig), implicating this population to be a facilitator of HIV infection.

Overall the majority of mucosal CD4^+^T cells expressing integrins, especially αEβ7^hi^CD4^+^T cells, were CCR5 positive, constituting potential targets to HIV infection (Fig 2C). The level of CCR5 expression in mucosal CD4^+^T cells expressing αEβ7, α4β7 or α4β1 warrants the investigation of targeting integrin-mediated migration or retention to reduce the availability of HIV target cells at the mucosa.

Therapeutically targeting integrin adhesion has already been used against autoimmune diseases such as inflammatory bowel disease (IBD), ulcerative colitis (UC) and multiple sclerosis (MS) and most recently gastrointestinal (GI) graft versus host disease [12, 13, 36]. Humanized monoclonal antibodies directed against the α4 subunit (Natalizumab) and against the α4β7 integrin (Vedolizumab) are among the therapies used in patients suffering with IBD, UC or MS. Vedolizumab has been the mAb of choice to block α4β7. Etrolizumab, a mAb that binds to β7 integrin subunit, can block α4β7-MAdCAM-1 and αEβ7-E-cadherin interactions and is being currently tested for the treatment of IBD and UC in clinical trials [37, 38].

The utilization of anti-α4β7 mAbs in NHP studies of HIV/SIV prevention and cure has shown promising results [14, 15, 39]. In Byrareddy et al (2016) animals treated with combined ART and anti-α4β7 mAb were able to sustain viral control for more than 2 years even after both therapies were withdrawn [15]. Although the mechanisms associated with this protection are unclear, the animals were able to restore T_H_17, T_H_22 and CD4^+^T_EM_ cells and displayed reduced plasma biomarkers associated with gut damage and inflammation [15]. Given the broad range of GI diseases where Vedolizumab has shown efficacy, there has been speculation that the relative protection in the viral controllers was mediated by reduced gut damage in the earliest phase of infection and thus preserving a functional immunological micro-environment.

The high levels of CD69 expression on αE^+^β7^hi^ T cells may indicate that these cells are tissue resident. Vaccine candidates that can promote and maintain specialized effector cells in the mucosa may offer a chance against challenging infectious agents, such as HIV [40–42]. It is conceivable to consider that pre-polarizing the mucosal immune response towards CD8^+^T_RM_ cells could have a protective effect against HIV. When therapeutically targeting integrin expression to modulate cell migration and retention it is important to consider that integrin expression by cells is dynamic, and targeting integrins such as α4β7 can potentially lead to a compensatory use of alternative integrins, such as αEβ7 and α4β1[24, 43]. Hence, understanding the regulatory mechanisms for integrins’ expression, the risk-benefits associated with anti-integrin blockade, and the contribution of each integrin for the migration and retention of a healthy CD8^+^:CD4^+^T cell ratio in the mucosa will help advancing towards better therapeutic and preventive strategies against infections such as HIV.

## Supporting information

S1 Fig Characterization of αE expression in distinct populations identified using anti-α4 and anti-β7 co-staining. (TIF)

S2 Fig Correlations between αE, β7 and α4 mean fluorescence intensities (MFI) in T cells isolated from blood, cervix and rectum. (TIF)

S3 Fig Density of β7, α4 and αE in integrin-expressing T cell subsets. (TIF)

S4 Fig Density of β7, α4 and αE in α4 and/or αE- expressing T cells. (TIF)
